# Invasiveness Does Not Predict Impact: Response of Native Land Snail Communities to Plant Invasions in Riparian Habitats

**DOI:** 10.1371/journal.pone.0108296

**Published:** 2014-09-19

**Authors:** Jitka Horáčková, Lucie Juřičková, Arnošt L. Šizling, Vojtěch Jarošík, Petr Pyšek

**Affiliations:** 1 Department of Ecology, Charles University in Prague, Faculty of Science, Prague 2, Czech Republic; 2 Department of Zoology, Charles University in Prague, Faculty of Science, Prague 2, Czech Republic; 3 Center for Theoretical Study, Charles University and the Academy of Sciences of the Czech Republic, Prague 1, Czech Republic; 4 Department of Invasion Ecology, Institute of Botany, Academy of Sciences of the Czech Republic, Průhonice, Czech Republic; University of Western Ontario, Canada

## Abstract

Studies of plant invasions rarely address impacts on molluscs. By comparing pairs of invaded and corresponding uninvaded plots in 96 sites in floodplain forests, we examined effects of four invasive alien plants (*Impatiens glandulifera*, *Fallopia japonica*, *F. sachalinensis*, and *F.×bohemica*) in the Czech Republic on communities of land snails. The richness and abundance of living land snail species were recorded separately for all species, rare species listed on the national Red List, and small species with shell size below 5 mm. The significant impacts ranged from 16–48% reduction in snail species numbers, and 29–90% reduction in abundance. Small species were especially prone to reduction in species richness by all four invasive plant taxa. Rare snails were also negatively impacted by all plant invaders, both in terms of species richness or abundance. Overall, the impacts on snails were invader-specific, differing among plant taxa. The strong effect of *I. glandulifera* could be related to the post-invasion decrease in abundance of tall nitrophilous native plant species that are a nutrient-rich food source for snails in riparian habitats. *Fallopia sachalinensis* had the strongest negative impact of the three knotweeds, which reflects differences in their canopy structure, microhabitat humidity and litter decomposition. The ranking of *Fallopia* taxa according to the strength of impacts on snail communities differs from ranking by their invasiveness, known from previous studies. This indicates that invasiveness does not simply translate to impacts of invasion and needs to be borne in mind by conservation and management authorities.

## Introduction

Invasive species are one of the major biotic stressors in native ecosystems all over the world [1]–[3], affecting the diversity of resident biota at various scales [4]–[10]. Plants are the most frequently studied group of invaders [11], [12] and in the last decades, extensive literature has accumulated on how they impact ecosystem structure, functioning and services [13]–[20].

The majority of studies on impacts of plant invasions focus on the same trophic level, i.e., what effects invasive species have on the performance of populations, species and communities of resident plants. In their global review of available data on impact, Pyšek et al. ([21]; their Table 1) found that effects of plant invasions on plant diversity are addressed about twice as frequently as those on animal diversity e.g., [22]–[25]; see [9], [26] for meta-analyses. However, invasive plants may alter interactions between trophic groups via the co-introduction of alien pollinators, seed dispersers, herbivores and predators, that cause profound disruptions to plant reproductive mutualisms [27], and by changing the biotic environment they may also impact reproductive output and population status of animal species [28], [29]. Invasions not only have major implications for biodiversity, but by forging novel functions in resident ecosystems, they also limit the effectiveness of restoration efforts that can be followed by unpredictable responses [18], [30].

**Table 1:** Quantitative summary of the effects of the four invasive plants studied on species numbers and abundances of land snail communities separated into groups (see text for criteria).

	Snail category					
Invading plant	Total		Small		Rare	
	Species number	Abundance	Species number	Abundance	Species number	Abundance
*Fallopia sachalinensis*	↓ 41.7	↓ 69.6	↓ 48.0			↓ 89.6
*F. japonica*			↓ 48.0			↓ 65.3
*F.×bohemica*			↓ 48.0			↑ 19.5
*Impatiens glandulifera*	↓ 16.3		↓ 48.0			↓ 28.8

Species numbers indicate percentage reduction in invaded compared to control plots, the arrow a decrease or increase in invaded plots. Empty cells refer to non-significant effects. Abundance is expressed as the number of living snail individuals.

Studies addressing the impacts of plant invasions on macroinvertebrates mostly reported significant reductions in species abundance, richness and diversity of arthropod communities [31]–[38] although in some studies this effect was restricted only to some groups [39]. Rarely, the studies reported shifting in food guilds [37]. However, studies exploring the impact of invasive plants on the abundance, species richness and diversity of molluscs, one of the model groups of herbivore generalists, are rather rare [40]–[46]. It has been shown that, for example, mollusc abundance decreased in areas invaded by *Tamarix ramosissima* in the southwestern United States [42] and in the riverine *Fallopia* stands in western Germany [43] and Switzerland [45]. Additionally, the litter of alien grasses from the genera *Avena* and *Bromus* reduced the number of snails in the Mediterranean biome of Australia [41]. However, the abundance of molluscs was not significantly affected in vegetation invaded by *Spartina anglica* in Australia [40] and both gastropod species richness and abundance even increased following invasion by *I. glandulifera* in northern Switzerland [46]. The results are thus rather scarce and contradictory and none of the studies compared the impact of several invasive plants on multiple criteria of mollusc performance. Such impacts are, however, likely to differ; mollusc assemblages were shown to respond strongly to the change in vegetation, with associated changes in calcium content and humidity being the most important factors determining their occurrence [46]–[48]. Therefore, the close dependence of land-snail assemblages on soil and vegetation, resulting from their food preferences, makes this group of invertebrates a promising model for studying the impact of plant invasion on higher trophic levels. It can be assumed that invasive plants differing in stature, canopy structure, and chemical composition of tissues would exert different impacts on the structure and composition of land-snail communities.

Here we examine the effects of four invasive alien plants on communities of land snails inhabiting invaded stands. The plants studied are all highly invasive in the Czech Republic [49] and include representatives of contrasting life forms: clonal perennials (three taxa of the genus *Fallopia*) versus an annual species (*I. glandulifera*). The impact of these invaders on plant diversity has been thoroughly documented (see below), but there is a lack of information on changes they induce in the species richness of terrestrial snail communities. To get insight into this issue we address the following questions: (1) Do invasive alien plants exert impacts on species richness and abundance of land snail communities? (2) If so, do the impacts differ with respect to particular invasive plant taxa? Finally, using the three *Fallopia* congeners for which there is a thorough knowledge of mechanisms of invasion in central Europe that makes it possible to rank them according to their invasiveness e.g., [50], [51], we ask (3) whether their ranking according to invasiveness corresponds to that based on the strength of impact on land snail communities?

## Materials and Methods

### Ethics statement

No permits and approvals were required for the field work, as sampling sites were under neither nature nor law protection.

### Invasive plants studied

*Fallopia japonica* (Houtt.) Ronse Decr. var. *japonica* and *F. sachalinensis* (F. Schmidt) Ronse Decr. (Polygonaceae) are stout rhizomatous perennials native to East Asia, introduced to Europe (the former as a single female clone that spread across the continent) as garden ornamentals and fodder plants in the 19th century [52], [53]. In the Czech Republic, both species are classified as invasive [10] and the genus *Fallopia* is represented also by the invasive hybrid *F.×bohemica* (Chrtek and Chrtková) J.P. Bailey, that is likely to have arisen on this continent several times independently and is also known from the native range of the parental species [53]. The first record of *F. japonica* var. *japonica* in the wild is from 1902, that of *F. sachalinensis* from 1921, and the earliest record of the hybrid *F.×bohemica* is from 1950. The invasion occurred in the second half of the 20th century, the hybrid lagged behind the two parental species but proceeded faster [54] due to its competitive superiority over the parents [50], [51]. In the early 2000s, *F. japonica* var. *japonica* was recorded from 1335 localities, *F. sachalinensis* from 261 and the hybrid from 382 [54]. Their dispersal is mainly vegetative through regeneration from rhizome and stem segments transported with contaminated soil and water [51], [55]. All three taxa became invasive (sensu [56], [57]) in a number of habitats including riparian, where they reach high covers and reduce species richness and diversity of invaded vegetation [49]. The invasion by *Fallopia* taxa exhibits the most severe impact on species richness and diversity among central-European alien plants, reducing the number of species present prior to invasion by 66–86%, depending on the taxon [23]. *Fallopia* taxa affect infrastructure by damaging roads and flood-prevention structures, and increasing the erosion potential of rivers [58], [59].

*Impatiens glandulifera* Royle (Balsaminaceae) is an annual species, up to 2.5 m tall, native to the Himalayas, introduced as a garden ornamental to Europe in 1839 and first recorded as escaped in 1855 [60]. In the Czech Republic, it was first recorded outside cultivation in 1896 [10], but rapid invasion only started in the mid-20th century [61]. *Impatiens glandulifera* is a dominant species of nitrophilous herbaceous fringes of rivers, willow galleries of loamy and sandy riverbanks and of riverine reed vegetation [49]. The species produces higher biomass than its congeners and is plastic in terms of response to nutrient availability and shading, but it also exhibits some genetically based population differentiation [62], [63]. Due to its massive spread and extensive populations in riparian habitats, it is considered a conservation problem [64]. However, despite forming populations with a high cover of up to 90%, it does not markedly reduce the numbers of species co-occurring in invaded stands, although invasion does alter species composition in favour of ruderal species [23], [65], but see [66]. *Impatiens glandulifera* was also shown to reduce the availability of pollinators for co-occurring native species [67].

The number of plant taxa included in the study was constrained by the fact that they represent a complete set of widespread invasive aliens in riparian habitats in the Czech Republic, with the only additional species being *Helianthus tuberosus*, that, however, invades different vegetation types than natural floodplain forests addressed in our study [49]. The massive invasion of all taxa under study on the rivers in the Czech Republic started at comparable times, around the mid-20^th^ century [61], [68], with some local differences [69]; in study sites the invasive species were permanently present for at least 10–15 years therefore it is unlikely that possible differences in residence times among localities affected the results.

### Study area and field sampling

Field work was conducted from 2006 to 2011. In total, 96 sites with a maximum distance of 279.5 km were located in floodplain forests, in alluvia of six rivers of the lower Elbe catchment area in the western part of the Czech Republic, Central Europe (see Supporting Information, Table S1). For each of the four invasive plant taxa, one pair of 10×10 m plots was established in each of the sampling sites (*I. glandulifera*, n = 16 paired plots at 32 sites; *F. japonica*, n = 10 paired plots at 20 sites; *F. sachalinensis*, n = 10 paired plots at 20 sites; *F.×bohemica*, n = 12 paired plots at 24 sites). One plot of the pair was located in invaded vegetation where the cover of the invader was 70–100%, the second non-invaded (control) plot was placed in a close vicinity to ensure that the habitat conditions matched as closely as possible to the invaded part [23]. Both plots in the studied pair were sampled once only on the same day. Most of the plots within pairs were placed within the distance of 200 m (median value 159 m) and with four exceptions, all plots were paired within one kilometre. In a few cases, the invader occurred in the non-invaded plot, but its cover range of 1–2% could not have any effect on vegetation or land snail species. That the invasion was the main factor in which plots within the pair differed was confirmed by direct measurements in both plots of the pair of environmental characteristics that might be important predictors of land snail species richness and composition [70], [71]. Of these we controlled for elevation (as a surrogate for climate), soil pH, and soil Ca content.

Land snail communities were sampled in the same plot as vegetation using a standard sampling procedure [72]. To document the presence of large and especially dendrophilous species (that rarely occur in litter samples), one person searched by eye for half an hour in all appropriate microhabitats within the whole plot, from which the litter sample could not be taken (e.g., dead wood, stones, tree trunks). Slugs were not included in data analysis because their activity depends mostly on weather conditions [73], and our sampling method was not suitable to record slugs quantitatively. The leaf litter samples with topsoil, twigs and vegetation were taken from four randomly selected quadrats (each measured 25×25 cm^2^) at each plot. These subsamples were amalgamated, air-dried and all shells were sorted out using sieves of different mesh size. All empty shells, including their fragments, were excluded from analyses in order to reduce potential bias caused by (1) including species that were not living in the locality but that had their shells redeposited by floods, (2) not including species living in the locality whose accumulated empty shells were removed by accidental flooding [74], and (3) a different length of shell degradation time in various floodplain forest types, which depends mainly on humidity [70], [75] and topsoil calcium content [76]. For these reasons in our analyses we only used the total numbers of living land snail species (further referred to as “total species”) and the total number of individuals per species. Species included in any of the four threat categories used in the Red List of molluscs of the Czech Republic [77] were labelled as “rare species” and considered as indicators of the state of molluscan assemblages. Species with shells smaller than 5 mm [78] were classified as “small species”. We distinguish this category because of biologically important variation of snail size in relationship to ecological [79] and geographical [80] scales. Specifically, large snails are often associated with moist conditions and low latitudes and thus their representation in communities is not uniform. Total number of species, the total number of individuals and their categorization into rare and small species, are shown in Table S2.

### Statistical analysis

Because the data were hierarchically structured in the sense that the invaded and non-invaded plots were nested within locations, to account for the spatial dependencies within locations statistical models were constructed by introducing a random effect for the locations. Invasion status of the plots (invaded/non-invaded) and plant taxon (*F. sachalinensis*, *F. japonica*, *F.×bohemica* and *I. glandulifera*; further termed ‘plant species’) were fixed factors and location a random intercept, implicitly introducing the compound symmetrical correlation structure [81]. To check whether the models adequately accounted for the spatial dependences in the data, meaning that the models did not violate the basic assumption of the independence of errors of the observations due to spatial autocorrelation [82], [83], we used a spline correlogram with 1000 resamples for bootstrap [84]–[86] based on Moran's *I* [87], [88], to investigate residuals of the models [89].

Numbers of total and small snail species were square-root or square-root+1 transformed, and numbers of total and small individuals log or log+1 transformed to normalize the data e.g., [90], and analyzed by linear mixed models (LMMs) using the function *lme* [91]. Numbers of rare species and individuals could not be transformed to normal distribution due to a large number of zero counts, and these data were therefore analyzed by generalized linear mixed models (GLMMs) with Poisson errors, using the functions *glmmPQL* [92] and *lmer* [93]. LMMs and the function *glmmPQL* also made it possible to calculate intra-class correlation, i.e., the associations between non-invaded and invaded plots within locations, and distinguish, after explaining the part of variance due to differences between the paired plots, the part of residual variance within paired plots at a particular location from the part of residual variance among the locations. Fitted models were checked by plotting appropriate residuals against fitted values and predictors, and by Q-Q plots e.g., [81]. Calculations were done in R 2.12.1 [94].

Finally, a binomial test across all snail species for all four invasive plant species was performed in order to assess the effect of the invaders on each snail species separately.

## Results

The effect of spatial autocorrelations was eliminated. This was so for all linear mixed models, and generalized linear mixed models obtained by application of *glmmPQL* function for rare species and *lmer* function for rare individuals (see Figure S1). This means that the explanatory variables were properly included in the models and their effects on the recorded mollusc species adequately measured, successfully accommodating for the spatial autocorrelation within the invaded and non-invaded plots.

High values of associations between non-invaded and invaded plots within sites, as well as relatively low residual variance within paired plots at each site and, at the same time, relatively high residual variance among sites (Table S3), indicate that the invaded and non-invaded plots within each site were appropriately selected. This is so because these results suggest that there was a relatively high similarity of the environmental factors listed above within the pairs of invaded and non-invaded plots within each site.

**Figure 1:**
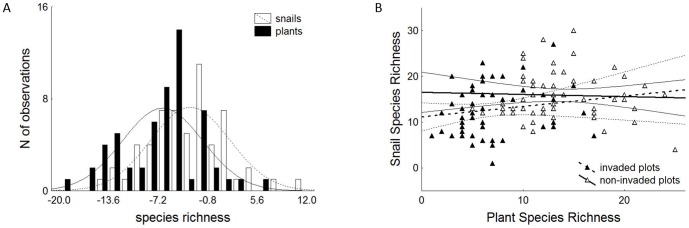
Frequency distribution of the pairwise residuals between species richness of invaded and non-invaded plots across the whole dataset. **A**) Frequency distribution of the pairwise residuals between species richness of invaded and non-invaded plots (richness of an invaded plot minus richness of the non-invaded plot) across the whole dataset. As the mean values of residuals in both the taxa (−6.7, and −3.1 for plants and snails, respectively) lies below zero value, and their two-sided 99% confidence intervals ([−8.5; −4.8] and [−4.9; −1.3], N = 58, df = 57) does not overlap zero, we conclude that the presence of the focal plant invaders reduce simultaneously plant and snail species richness. **B**) The lack of significant relationship (straight lines – mean trends, curved lines 95% confidence intervals) between the plant and snail species richness (in both the invaded-full lines, open symbols- and non-invaded plots-dashed lines, full symbols- as well as when using pooled data, where all the plots were analyzed together-is not shown) suggests that the reduction of plant species richness at the invaded plots is not a direct driver of the observed snail species richness reduction.

A simple pairwise test indicated significant reduction of plant and snail species richness at the invaded sites (p<0.01, df = 57, for details see legend to the Fig. 1A), but none or insignificant difference in elevation, soil pH, and soil Ca content between the invaded and non-invaded sites (Fig. S2). Moreover the snail species richness was independent from plant species richness in both the invaded and non-invaded plots as well as when using pooled data, where all the plots were analyzed together (Fig. 1B). Hence we conclude that neither the reduction of plant species richness nor difference in environmental factors between the invaded and non-invaded sites can be a direct driver of snail species richness. We therefore interpret the reduction of snail species richness as a consequence of the focal plant invaders presence/absence at the sites.

**Figure 2:**
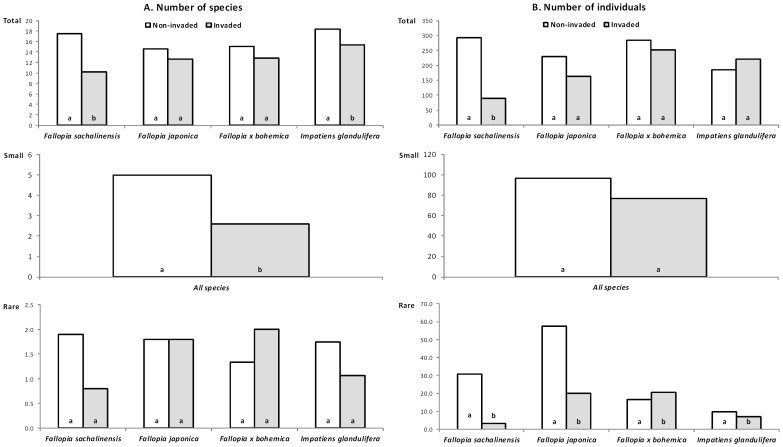
Average numbers of snail species and individuals from 48 paired invaded and non-invaded plots. Average numbers of total, small and rare snail species (A) and individuals (B) from 48 paired plots at individual sites for the species of invasive plants studied (*Fallopia sachalinensis*, *F. japonica*, *F.×bohemica* and *Impatiens glandulifera*). Counts for small snail species and individuals are shown together for all invasive plants as these numbers changed consistently for all plant species (non-significant invasion status×plant species interaction in Table 2). Paired columns followed by different letters differ significantly (*P*<0.05). Full statistics are given in Table 4.

Except for small snails, the effect of individual plant taxa on the numbers of all snail species (i.e., including small and rare) and individuals was statistically different, as indicated by the significant plant species×invasion status interactions (Tables 2 and 3). *Fallopia sachalinensis* had the greatest negative effect on snail communities, significantly decreasing the total number of species and individuals, and the number of rare individuals. *Impatiens glandulifera* had a significant negative effect on the total number of species and on rare individuals. *Fallopia japonica* significantly decreased the number of rare individuals. Surprisingly, *F.×bohemica* significantly increased the number of rare individuals. All invasive species had the same, significant negative effect on the number of small snail species (Fig. 2, Tables 1 and 4).

**Table 2:** ANOVA tables for the numbers of total and small snail species and individuals.

Source of variation	Species						Individuals					
	Total			Small			Total			Small		
	Df	F	P	Df	F	P	Df	F	P	Df	F	P
Site status	1, 44	25.707	<0.0001	1, 44	6.589	<0.05	1, 44	8.181	<0.01	1, 44	3.159	<0.1
Plant species	3, 44	1.499	NS	3, 44	0.935	NS	3, 44	1.129	NS	3, 44	0.207	NS
Status×Plant	**3, 44**	**3.115**	**<0.05**	3, 44	1.640	NS	**3, 44**	**6.800**	**<0.001**	3, 44	1.844	NS

ANOVA tables for the numbers of total and small snail species and individuals, analyzed by linear mixed models with plot invasion status (invaded/non-invaded) and invading plant species (*Fallopia sachalinensis*, *F. japonica*, *F.×bohemica* and *Impatiens glandilifera*) as fixed effects and sites with paired invaded/non-invaded plots as random intercepts. Significant invasion status×plant species interactions are in bold. Rare species and individuals were analyzed by generalized linear models (GLMMs) for which ANOVAs are not available. Results of t-tests for the fixed effects of these GLMMs are in Table 3.

**Table 3:** Results of t-tests for the numbers of rare snail species and individuals.

Source of variation	Rare species					Rare individuals			
	Value	Std. Error	Df	t-value	P	Estimate	Std. Error	z-value	P
Intercept	0.144	0.294067	44	0.489	NS	**2.0096**	**0.484**	**4.154**	**<0.001**
Status invasive	0.405	0.242285	44	1.674	NS	**0.2171**	**0.096**	**2.273**	**<0.05**
*Fallopia japonica*	0.257	0.424453	44	0.607	NS	1.1191	0.713	1.569	NS
*Fallopia sachalinensis*	0.270	0.425662	44	0.634	NS	−0.1536	0.733	−0.209	NS
*Impatiens glandulifera*	0.204	0.382983	44	0.534	NS	−0.4884	0.646	−0.757	NS
Invasive*×Fallopia japonica*	−0.405	0.348307	44	−1.164	NS	**−1.2764**	**0.126**	**−10.126**	**<0.001**
Invasive×*Fallopia sachalinensis*	**−1.270**	**0.398501**	**44**	**−3.188**	**<0.01**	**−2.4814**	**0.209**	**−11.875**	**<0.001**
Invasive×*Impatiens glandulifera*	**−0.90446**	**0.33462**	**44**	**−2.70294**	**<0.01**	**−0.5574**	**0.157**	**−3.556**	**<0.001**

Results of t-tests for the numbers of rare snail species and individuals, analyzed by generalized linear mixed models with plot invasion status (invaded/non-invaded) and invading plant species (*Fallopia sachalinensis*, *F. japonica*, *F.×bohemica* and *Impatiens glandulifera*) as fixed effects and sites with paired invaded/non-invaded plots as random intercepts. Rare species were analyzed using the function *glmmPQL* and rare individuals using *lmer* in R [94]. Results were obtained by the function *summary* and show fixed effects based on treatment contrasts where the intercept is for the plot invasion status ‘non-invaded’ and invading plant species *F.×bohemica*. Significant invasion status×plant species interactions are in bold.

**Table 4:** Results of full statistical analyses describing numbers of total, small and rare snail species and individuals between non-invaded and invaded plots.

Numbers of species	Total						Small						Rare					
	Difference			F	Df	P	Difference			F	Df	P	Difference			t-value	Df	P
*Fallopia sachalinensis*	**−1.070**	**±0.189**	**(−7.3)**	**32.519**	**1, 9**	**<0.001**	**−0.229**	**±0.091**	**(−2.4)**	**6.332**	**1, 47**	**< 0.05**	−0.865	±0.438	(−1.1)	1.473	9	NS
*Fallopia japonica*	−0.298	±0.256	(−2.0)	1.350	1, 9	NS	**−0.229**	**±0.091**	**(−2.4)**	**6.332**	**1, 47**	**< 0.05**	0.000	±0.150	(0.0)	0.000	9	NS
*Fallopia×bohemica*	−0.315	±0.177	(−2.3)	3.176	1, 11	NS	**−0.229**	**±0.091**	**(−2.4)**	**6.332**	**1, 47**	**<0.05**	0.405	±0.206	(0.7)	1.964	11	NS
*Impatiens glandulifera*	**−0.390**	**±0.169**	**(−3.0)**	**5.359**	**1, 15**	**<0.05**	**−0.229**	**±0.091**	**(−2.4)**	**6.332**	**1, 47**	**<0.05**	−0.499	±0.236	(−0.7)	2.117	15	NS

Numbers of individuals	Total						Small						Rare				
	Difference			F	Df	P	Difference			F	Df	P	Difference			z-value	P
*Fallopia sachalinensis*	**−1.408**	**±0.309**	**(−203.9)**	**20.821**	**1, 9**	**<0.001**	−0.437	±0.252	(−17.3)	2.998	1, 47	< 0.1	**−2.264**	**±0.186**	**(−27.6)**	**12.185**	**<0.001**
*Fallopia japonica*	−0.556	±0.367	(−66.3)	2.291	1, 9	NS	−0.437	±0.252	(−17.3)	2.998	1, 47	<0.1	**−1.059**	**±0.082**	**(−37.5)**	**12.876**	**<0.001**
*Fallopia×bohemica*	−0.188	±0.250	(−31.5)	0.568	1, 11	NS	−0.437	±0.252	(−17.3)	2.998	1, 47	<0.1	**0.217**	**±0.095**	**(4)**	**2.273**	**<0.05**
*Impatiens glandulifera*	0.229	±0.186	(36.2)	1.526	1, 15	NS	−0.437	±0.252	(−17.3)	2.998	1, 47	<0.1	**−0.340**	**±0.124**	**(−2.8)**	**2.74**	**<0.01**

Results of full statistical analyses describing numbers of total, small and rare snail species and individuals between non-invaded plots and plots invaded by plant species *Fallopia sachalinensis*, *F. japonica*, *F.×bohemica* and *Impatiens glandulifera*. Difference is a change in counts between non-invaded and invaded plots, with negative values indicating a decrease in snail numbers on invaded sites. Values ± standard errors are on transformed scales, numbers in parentheses are original counts. Significant differences between invaded and non-invaded plots for the individual plant species are in bold.

Detailed binomial analyses showed, after the Bonfferoni correction applied at the significance level of 0.01, that *I. glandulifera* decreased abundances of 11 species and increased those of eight species of the 51 in total; *F.×bohemica* decreased and increased abundances of 11 and six species, respectively, of 54 in total; *F. japonica* decreased abundances of seven species and increased abundances of two species of 50 in total; and *F. sachalinensis* decreased abundances of 19 of 43 species. The snails whose abundances were significantly higher at sites with the invasive plant present compared to non-invaded ones belonged mostly to small, leaf litter-dwelling species (see Table S4).

## Discussion

### Impacts on snail communities are invader-specific

Our study shows that invasive plants in temperate riparian habitats significantly affect species composition and structure of land-snail communities, and that these impacts vary with respect to the ecological groups of snails (i.e., they depend on woodland, open-country, mesic and/or aquatic character of particular snail species). Overall, the significant impacts range from 16 to 48% reduction in terms of species numbers, and 29–90% reduction in abundance. However, unlike in previous studies that mostly addressed the impacts of a single invasive plant species on mollusc communities e.g., [40], [42], [43], [45], [46], but see [41], our results provide insights into how impacts differ with respect to the identity of the invader.

That *I. glandulifera* was the plant with the second strongest impact on snail communities in the study, the only one besides *F. sachalinensis* that decreased total snail numbers, is rather surprising. This plant forms less homogenous and less dense cover than *Fallopia* taxa, and was reported to exert relatively minor impact on species richness of native plants following invasion in the Czech Republic [23], [65]. This plant species has been documented to cause an increase in the richness and abundance of gastropods in deciduous forests in Switzerland, attributed to higher humidity in invaded sites [46]. It needs to be noted, however, a greater impact on native plant species richness than that recorded in the Czech Republic was reported from the UK [22]. The strong effect of this invader could be related to the decreased abundance, following invasion, of tall nitrophilous native species (e.g., *Urtica dioica* and *Aegopodium podagraria*) that are a nutrient-rich food source for snails in riparian habitats. *Urtica*-dominated stands are characteristic of the understory of native floodplain forests in the study area, and harbour typical woodland snail fauna that includes a number of rare species.

*Fallopia sachalinensis* had the strongest negative impact on land-snail communities. Invasion by this species markedly decreased total species number and abundance of snails, as well as the number of small species and abundance of rare species. This is in contrast with the recorded impacts of the other two *Fallopia* taxa, which were much less profound and did not affect the snail community as a whole; their effects were only evident with regard to small and/or rare species. Interestingly, Stoll et al. [45] who examined the impact of a single *Fallopia* species, *F. japonica*, on snail communities in northern Switzerland arrived to opposite conclusions. This invasion reduced average snail richness but the impact differed with respect to shell size; the decreases in species richness were even more pronounced in large, long-lived species as compared to slugs and small, short-lived snails. Moreover, in our study there was a positive effect of *F.×bohemica*, *F. japonica*, and *I. glandulifera* on numbers of individuals of rare snail species. Nevertheless, the overall pattern of impacts markedly differing among the three closely related taxa is surprising if compared to how they affect the plant species richness of invaded communities. The degree to which plant species richness is reduced following invasion is rather high and consistent for all three *Fallopia* taxa. They exhibit one of the most severe impacts on species richness and diversity among central-European alien plants, reducing the number of species present prior to invasion by 66–86%, depending on the taxon [23]; see also [45] for *F. japonica*.

Laboratory experiments may shed a light on the differences among the three *Fallopia* taxa in respect with the impact on snail communities. Laboratory experiments have shown that there exists a pronounced phytotoxic effect of *Fallopia* leaf extracts on seed germination. *Fallopia sachalinensis* exerts the largest negative effect on germination of *Urtica dioica*, the most abundant native species commonly growing in floodplain habitats invaded by *Fallopia* taxa in the studied area, while *F.×bohemica* consistently has the lowest inhibitory effect [97]. Although these results do not provide direct evidence for differential effect of the individual *Fallopia* taxa on snail communities, they clearly show that litter quality differs among the *Fallopia* species and their hybrid. Importantly, this difference in phytotoxicity of leaf litter for seed germination is consistent with the different impact of the individual *Fallopia* taxa on snail community; *F. sachalinensis* had consistently the strongest negative impact on land snail communities, while for *F.×bohemica* there was a positive effect. In addition, the high negative effect of *F. sachalinensis* leaf litter on germination of native plant species can further exacerbate the negative effect of this species on snail communities by an indirect way, via the suppression of the important native food plant *U. dioica*.

### Body size affects the response of snails to invasion

Only snail community characteristics for which the impact was not invader-specific is the proportion of small species relative to the total snail community. Our results indicate that small snail species are a group especially prone to reduction in species richness resulting from plant invasions; their numbers in invaded plots consistently decreased regardless of the identity of the invader (but see [45]). This holds also for plots invaded by *F. japonica* and *F.×bohemica*, where total snail numbers were not affected, but the proportion of small snails decreased by 48% (Table 1). Stoll et al. [45] argued that small snail species in *F. japonica* invaded plots feed on algae, fungi and leaf litter, hence are less impacted by invasion than herbivorous large snails suffering from low palatability of knotweed tissues caused by high concentrations of phenolic compounds and lignin [96], [97]. On the other hand, slow decomposition of knotweed litter [97], [98] most likely results in a limited availability of food for small snail species, causing their reduction in invaded plots.

The post-invasion shift in snail species size hierarchies can be also linked to large snail species controlling a greater proportion of available resources than the smaller ones [80]. It can be hypothesized that under deteriorated conditions and namely reduced diversity of available food after the invasions, small snails are more affected than large ones that are superior in utilization of the limited resources [99]. An additional explanation could be that large snails inhabiting riparian vegetation are capable of profiting from the presence of tall invasive plants due to their climbing behaviour which is not the case of epigeic small snails.

### High invasiveness does not automatically translate into strong impact

The three *Fallopia* taxa addressed in our study represent a thoroughly investigated study system for which there is detailed information on the history of invasion, ecology and traits conferring invasiveness in the invaded range in Europe. Previous research consistently points to an increased invasiveness of the hybrid compared to both parental species. The hybrid was reported to spread faster [54], and its abundance in the landscape can be related to better regeneration capacity from rhizome fragments [51]; it is also more difficult to control [50], [55] and was a superior competitor to both parents when grown together in an experimental garden (P. Pyšek et al. unpublished data). In other studies, one of the parents performed poorly, such as with *F. sachalinensis* in a field study addressing the establishment of the three taxa [100] or *F. japonica* in a laboratory study investigating phytotoxic effects on germination of native species [95]; however, in both studies the hybrid was, together with the other parent, superior to the poorly performing one.

That the ranking of *Fallopia* taxa according to the strength of impacts on snail communities markedly differs from that according to their invasiveness as measured in the above studies, points to the fact that invasiveness does not simply translate to impacts. This is in accordance with conclusions of Ricciardi and Cohen [7] who found no correlations between invasiveness of alien plants, mammals, fishes, invertebrates, amphibians and reptiles, and their impact on biodiversity on a broad scale. Although the issue requires further study the possibility that the mechanisms of invasion and impact may not be strongly linked needs to be taken into account by managers. For our study it needs to be borne in mind that the impact on snail communities is only one particular type of impacts of plant invasions. Therefore, our results also emphasize the necessity of employing a variety of response measures when studying impacts of invasive species, as what we measure to a large extent determines whether or not the impact of a particular invasion appears serious [21], [20].

The results of our study convey an important message for conservation authorities in the Czech Republic. Riparian habitats serve as refugia for many snail species that lost the majority of their natural habitats in the fragmented, intensively used landscape. Invasions of riparian zones by alien plants are an important factor further contributing to deterioration of snail habitats, and knotweeds are among the major invaders of these habitats. Focusing management effort on the hybrid, as the taxon with the greatest potential to spread [54], and paying the least widespread parent, *F. sachalinensis*, less attention, would be justified if one was primarily concerned with plant diversity. Without knowledge of impacts on snails, as documented in our study, this might seem the best strategy in general. However, based on a more comprehensive picture of taxon-specific impacts that vary with respect to the affected group of biota, and with specific conservation goals in mind, our results may help to inform conservation policy in a given area. For example, in regions with high land snail diversity and conservation value, allocation of resources to *Fallopia* control should reflect the ranking of taxa according to impact on snails.

## Supporting Information

Figure S1 **Spline autocorrelation statistics for residuals of models describing the numbers of total, small and rare snail species and individuals.**
(DOC)

Figure S2 **Frequency distribution of residuals between environmental parameters of the invaded and non-invaded plots.**
(DOCX)

Table S1 **Overview of non-invaded and invaded sites used in this study.**
(DOC)

Table S2 **Overview of all recorded land snail species.**
(DOC)

Table S3 **Results of full statistical analyses describing intraclass correlations between plots and partition of residual variance for numbers of total, small and rare snail species and individuals between invaded and non-invaded plots.**
(DOC)

Table S4 **Results of binomial analyses describing differences in total abundances of each snail species in the invaded and non-invaded plots.**
(DOC)
