# Correction: Invasiveness Does Not Predict Impact: Response of Native Land Snail Communities to Plant Invasions in Riparian Habitats

**DOI:** 10.1371/journal.pone.0114751

**Published:** 2014-12-01

**Authors:** The PLOS ONE Staff

The following information is missing from the Funding section: This work was also supported by Czech Science Foundation no. P505/11/2387.
