# Supplementary material for: Invasiveness Does Not Predict Impact: Response of Native Land Snail Communities to Plant Invasions in Riparian Habitats
Source: PLoS One. 2014 Sep 19;9(9):e108296. doi: 10.1371/journal.pone.0108296 (PMC4169606; doi:10.1371/journal.pone.0108296)
Supplement: Figure S1 — Spline autocorrelation statistics for residuals of models describing the numbers of total, small and rare snail species and individuals. (DOC) [file pone.0108296.s001.doc]

**Figure S1. Spline autocorrelation statistics for residuals of models describing the numbers of total, small and rare snail species and individuals.**

**
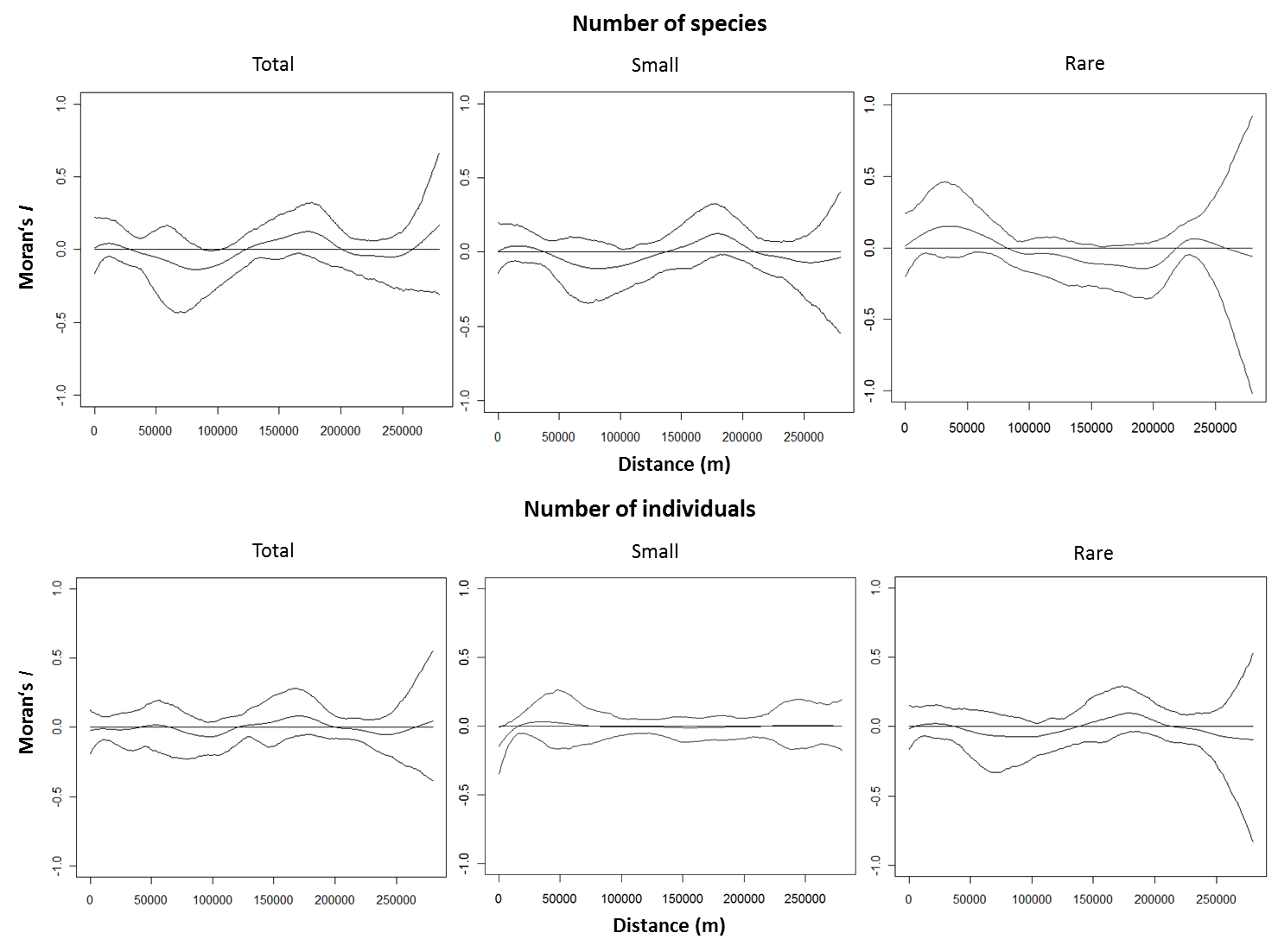
**

Spline autocorrelation statistics for residuals of models describing the numbers of total, small and rare snail species and individuals, analyzed based on invasion status of plots (invaded/non-invaded) and invading plant species (*Fallopia sachalinensis*, *F. japonica*, *F. ×bohemica* and *Impatiens glandulifera*) as fixed, and locations with the paired plots as random effects. The statistics are expressed as Moran’s *I* correlograms plotted against continuous functions of distance (m) of the examined plots. *I*’*s* (mid line) vary between +1 and –1, having expected value near zero for no spatial autocorrelation, with negative and positive values indicating negative and positive autocorrelation, respectively. No spatial autocorrelation means that data collated from the sites and the plots within sites at different distances apart do not exhibit more similar/dissimilar records than expected by chance. Significant positive/negative autocorrelations would appear as the upper and lower lines, describing 95% pointwise bootstrap confidence intervals, both above/below the zero line. Results for total and small species and individuals are based on linear mixed models (LMMs), and results for rare species and individuals based on generalized linear mixed models (GLMMs) fitted by *glmmPQL* function (number of species) and *lmer* function (number of individuals). See Table 2 for ANOVA tables of LMMs, Table 3 for t-test of GLMMs, and Table 4 for full statistics of all models.
