# Supplementary material for: Invasiveness Does Not Predict Impact: Response of Native Land Snail Communities to Plant Invasions in Riparian Habitats
Source: PLoS One. 2014 Sep 19;9(9):e108296. doi: 10.1371/journal.pone.0108296 (PMC4169606; doi:10.1371/journal.pone.0108296)
Supplement: Figure S2 — Frequency distribution of residuals between environmental parameters of the invaded and non-invaded plots. (DOCX) [file pone.0108296.s002.docx]

**Figure S2. Frequency distribution of residuals between environmental parameters of the invaded and non-invaded plots.**


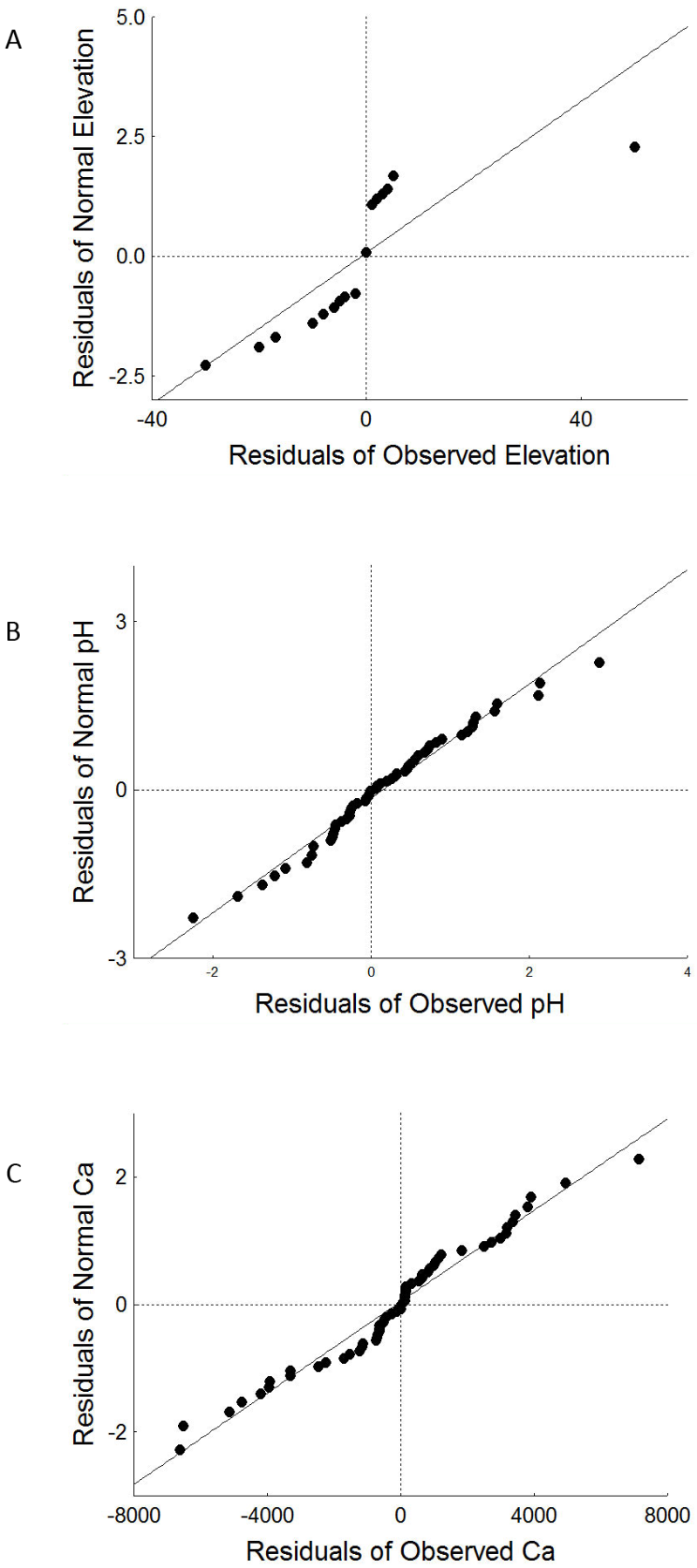


Normal probability plots show frequency distribution of residuals between environmental parameters of the invaded and non-invaded plots (A-elevation as a proxy of a climate; B-soil pH; C-soil Ca). Full line is a picture distribution had it been perfectly normal. Dotted lines show that all the distributions attain zero values for the observed and expected residuals which means no bias toward higher parameters of the invaded or non-invaded plots. The facts that app the same numbers of points lie above and below zero and 95% confidence intervals of the mean values cover the zero value ([-3.4, 1.4], [-0.1, 0.4], [-830, 580], respectively; df=57) indicate no difference or insignificant difference. (N=58, some points, however, overlap each other as many values are identical and thus the values [0,0] are plotted against each other.)
