# Supplementary material for: Invasiveness Does Not Predict Impact: Response of Native Land Snail Communities to Plant Invasions in Riparian Habitats
Source: PLoS One. 2014 Sep 19;9(9):e108296. doi: 10.1371/journal.pone.0108296 (PMC4169606; doi:10.1371/journal.pone.0108296)
Supplement: Table S1 — Overview of non-invaded and invaded sites used in this study. (DOC) [file pone.0108296.s003.doc]

**Table S1.** **Overview of non-invaded and invaded sites used in this study.**
