# Supplementary material for: Invasiveness Does Not Predict Impact: Response of Native Land Snail Communities to Plant Invasions in Riparian Habitats
Source: PLoS One. 2014 Sep 19;9(9):e108296. doi: 10.1371/journal.pone.0108296 (PMC4169606; doi:10.1371/journal.pone.0108296)
Supplement: Table S2 — Overview of all recorded land snail species. (DOC) [file pone.0108296.s004.doc]

**Table S2.** **Overview of all recorded land snail species.**

**Table S2.** Continued

Overview of all recorded living land snail species, their frequency of occurrence and numbers of individuals per invaded/non-invaded plots, and their categorization into: r = rare species, and s = small species. Note that all snails recorded are native with one exception of three individuals of *Oxychilus draparnaudi* in *Fallopia ×bohemica* stand; this species is native to western Europe and long naturalizated in the Czech Republic.
