# Supplementary material for: Invasiveness Does Not Predict Impact: Response of Native Land Snail Communities to Plant Invasions in Riparian Habitats
Source: PLoS One. 2014 Sep 19;9(9):e108296. doi: 10.1371/journal.pone.0108296 (PMC4169606; doi:10.1371/journal.pone.0108296)
Supplement: Table S3 — Results of full statistical analyses describing intraclass correlations between plots and partition of residual variance for numbers of total, small and rare snail species and individuals between invaded and non-invaded plots. (DOC) [file pone.0108296.s005.doc]

**Table S6.** **Results of full statistical analyses describing intraclass correlations between plots and partition of residual variance for numbers of total, small and rare snail species and individuals between invaded and non-invaded plots.**

Intraclass correlations (ICC) between paired non-invaded plots and plots invaded by plant species *Fallopia sachalinensis*, *F. japonica*, *F.* ×*bohemica* and *Impatiens glandulifera*, and variance calculated after explaining the part of variance due to differences between the paired plots. Variance ”between” is a residual variance between sites with the paired plots, and variance “within” a residual variance within paired plots. The ICC and variance values could not be calculated for numbers of rare individuals because they were analyzed by generalized linear mixed models using the function *lmer* that does not enable their assessment.
