# Supplementary material for: Invasiveness Does Not Predict Impact: Response of Native Land Snail Communities to Plant Invasions in Riparian Habitats
Source: PLoS One. 2014 Sep 19;9(9):e108296. doi: 10.1371/journal.pone.0108296 (PMC4169606; doi:10.1371/journal.pone.0108296)
Supplement: Table S4 — Results of binomial analyses describing differences in total abundances of each snail species in the invaded and non-invaded plots. (DOC) [file pone.0108296.s006.doc]

**Table S7.** **Results of binomial analyses describing differences in total abundances of snail species in the invaded and non-invaded plots.**

|  |  | *Imp-gla* |  | *Fall-boh* |  | *Fall-jap* |  | *Fall-sach* |
| --- | --- | --- | --- | --- | --- | --- | --- | --- |
| Species name |  | *n* = 51 |  | *n* = 54 |  | *n* = 50 |  | *n* = 43 |
| *Acanthinula aculeata* |  | 2,91E-01 |  | 2,89E-01 |  | 3,90E-01 | ↓ | **1,37E-17** |
| *Aegopinella minor* |  |  |  | 1,56E-02 |  | 6,25E-02 |  | 4,88E-04 |
| *Aegopinella nitens* | ↓ | **2,32E-10** |  | 2,50E-01 |  | 3,91E-03 |  |  |
| *Aegopinella nitidula* |  | 1,56E-02 | ↓ | **5,03E-08** |  | 1,16E-01 | ↓ | **2,83E-08** |
| *Aegopinella pura* |  | 3,59E-01 | ↑ | **4,38E-05** | ↓ | **9,69E-06** |  | 8,25E-03 |
| *Aegopinella ressmanni* |  |  |  | 4,30E-01 |  |  |  |  |
| *Aegopis verticillus* |  |  |  | 7,30E-02 |  |  |  |  |
| *Alinda biplicata* | ↓ | **1,08E-11** |  | 4,77E-01 | ↓ | **8,20E-11** |  | 4,26E-03 |
| *Arianta arbustorum* | ↑ | **6,49E-05** |  | 2,69E-01 |  | 6,76E-02 |  | 1,26E-01 |
| *Carychium minimum* | ↓ | **6,17E-08** |  | 1,18E-01 |  | 7,40E-02 | ↓ | **1,82E-09** |
| *Carychium tridentatum* | ↓ | **2,87E-07** | ↑ | **1,11E-27** |  | 5,09E-02 |  | 3,53E-03 |
| *Cepaea hortensis* |  | 3,87E-01 |  | 4,81E-02 |  | 4,19E-01 |  | 5,47E-02 |
| *Cepaea nemoralis* |  |  |  |  |  | 2,54E-01 |  |  |
| *Clausilia bidentata* |  |  |  |  |  | 5,93E-01 |  |  |
| *Clausilia pumila* |  | 8,89E-02 |  | 1,72E-01 |  | 1,25E-01 |  |  |
| *Cochlicopa lubrica* |  | 5,27E-02 | ↑ | **7,80E-10** |  | 3,90E-01 | ↓ | **5,40E-84** |
| *Cochlodina corcontica* |  |  |  |  |  | 1,25E-01 |  |  |
| *Cochlodina laminata* | ↓ | **1,62E-04** |  | 3,13E-01 |  | 4,61E-02 |  | 4,88E-04 |
| *Columella aspera* |  |  |  |  |  |  |  | 7,50E-01 |
| *Columella edentula* |  | 4,03E-02 | ↓ | **6,78E-06** |  | 1,35E-02 | ↓ | **3,92E-10** |
| *Daudebardia brevipes* |  | 1,07E-02 |  | 3,44E-01 |  | 3,13E-02 |  | 3,44E-01 |
| *Daudebardia rufa* |  | 1,71E-03 |  | 1,17E-03 |  | 6,36E-03 |  |  |
| *Discus perspectivus* | ↑ | **1,80E-08** |  |  |  |  |  |  |
| *Discus rotundatus* | ↑ | **2,83E-14** |  | 1,45E-01 |  | 2,54E-03 | ↓ | **1,01E-05** |
| *Ena montana* | ↓ | **7,25E-05** |  | 1,72E-01 |  | 8,98E-02 |  | 2,50E-01 |
| *Eucobresia diaphana* |  | 1,12E-02 |  | 1,30E-03 |  | 7,81E-03 |  | 4,08E-02 |
| *Euconulus fulvus* |  | 6,05E-01 |  | 3,83E-04 | ↑ | **5,12E-09** |  | 1,09E-01 |
| *Euconulus praticola* |  |  |  | 3,63E-01 |  |  |  | 2,50E-01 |
| *Fruticicola fruticum* |  | 1,49E-01 | ↓ | **3,68E-55** |  | 1,22E-02 | ↓ | **2,37E-07** |
| *Helix pomatia* |  | 3,44E-01 |  | 2,74E-01 |  | 1,19E-01 |  | 7,81E-03 |
| *Isognomostoma isognomostomos* |  | 4,34E-01 |  | 1,88E-01 |  | 1,93E-02 |  | 1,71E-03 |
| *Laciniaria plicata* |  | 7,81E-03 |  | 5,00E-01 |  |  |  |  |
| *Macrogastra plicatula* | ↓ | **2,98E-08** | ↑ | **1,36E-04** |  | 1,63E-01 |  | 6,25E-02 |
| *Macrogastra tumida* |  | 2,76E-01 |  |  |  |  |  |  |
| *Macrogastra ventricosa* |  | 2,54E-01 |  | 3,13E-02 |  | 5,47E-02 |  |  |
| *Monachoides incarnatus* |  | 6,11E-03 |  | 5,00E-01 |  | 6,93E-02 | ↓ | **1,26E-07** |
| *Oxychilus alliarius* |  | 2,50E-01 |  |  |  |  |  |  |
| *Oxychilus cellarius* |  | 3,63E-01 |  | 5,00E-01 |  | 2,15E-01 |  | 2,50E-01 |
| *Oxychilus depressus* |  | 5,00E-01 |  |  |  |  |  |  |
| *Oxychilus draparnaudi* |  |  |  | 1,25E-01 |  |  |  |  |
| *Oxychilus glaber* |  |  |  | 1,25E-01 |  |  |  | 2,71E-01 |
| *Perforatella bidentata* |  | 5,00E-01 |  |  | ↓ | **5,32E-50** |  | 5,00E-01 |
| *Perpolita hammonis* | ↑ | **4,11E-14** | ↓ | **2,10E-17** |  | 9,62E-04 | ↓ | **5,35E-24** |
| *Perpolita petronella* |  | 7,81E-03 |  |  |  | 3,13E-02 |  |  |
| *Petasina unidentata* |  | 2,50E-01 |  |  |  |  |  | 5,00E-01 |
| *Pseudotrichia rubiginosa* | ↓ | **1,53E-04** | ↓ | **7,69E-05** | ↓ | **9,66E-13** | ↓ | **1,93E-25** |
| *Punctum pygmaeum* |  | 3,43E-01 | ↓ | **6,76E-18** | ↑ | **3,07E-08** | ↓ | **6,66E-14** |
| *Ruthenica filograna* |  | 3,13E-02 |  |  | ↓ | **3,05E-05** | ↓ | **3,47E-18** |
| *Semilimax semilimax* |  | 3,44E-01 |  | 7,30E-02 |  | 8,98E-02 |  | 1,05E-03 |
| *Succinea putris* | ↑ | **9,08E-59** |  | 2,50E-04 | ↓ | **7,42E-05** |  | 5,00E-01 |
| *Succinella oblonga* |  | 1,09E-01 | ↓ | **1,62E-04** |  | 3,77E-03 |  | 4,88E-04 |
| *Trochulus hispidus* |  | 1,98E-02 |  | 2,61E-01 |  | 8,47E-04 | ↓ | **2,51E-12** |
| *Trochulus sericeus* |  | 1,97E-03 |  | 3,91E-03 |  | 3,91E-03 | ↓ | **6,10E-05** |
| *Truncatellina cylindrica* |  |  |  | 1,25E-01 |  |  |  |  |
| *Urticicola umbrosus* | ↑ | **3,22E-09** | ↓ | **2,89E-08** |  | 3,92E-01 | ↓ | **1,53E-06** |
| *Vallonia costata* |  | 1,58E-02 | ↓ | **3,30E-57** | ↓ | **1,42E-10** |  |  |
| *Vallonia pulchella* |  |  |  | 2,40E-01 |  | 1,48E-01 | ↓ | **2,30E-41** |
| *Vertigo alpestris* |  |  |  | 2,50E-01 |  |  |  |  |
| *Vertigo angustior* |  |  | ↓ | **6,10E-05** |  |  |  |  |
| *Vertigo pusilla* | ↓ | **7,62E-06** | ↑ | **1,49E-08** |  | 1,14E-01 | ↓ | **1,30E-13** |
| *Vertigo pygmaea* |  | 2,50E-01 | ↓ | **6,10E-05** |  |  |  |  |
| *Vertigo substriata* |  |  |  |  |  | 5,00E-01 |  |  |
| *Vitrea contracta* | ↓ | **6,10E-05** |  |  |  | 6,25E-02 |  |  |
| *Vitrea crystallina* | ↑ | **9,50E-14** | ↑ | **4,18E-12** |  | 2,49E-03 |  | 2,72E-01 |
| *Vitrea diaphana* |  |  |  | 6,88E-01 |  |  |  |  |
| *Vitrea subrimata* |  |  |  | 5,00E-01 |  |  |  |  |
| *Vitrina pellucida* | ↓ | **5,80E-10** |  | 5,00E-01 |  | 1,22E-02 | ↓ | **1,65E-14** |
| *Zonitoides nitidus* | ↑ | **8,56E-38** |  | 3,67E-03 |  | 3,30E-03 | ↓ | **3,87E-24** |

The Bonfferoni correction was applied on significance level 0.01. The arrows highlight significant decrease (↓) or increase (↑) of each snail species in invaded plots.
